# A Single Center Retrospective Cohort Study Comparing Different Anticoagulants for the Treatment of Catheter-Related Thrombosis of the Upper Extremities in Women With Gynecologic and Breast Cancer

**DOI:** 10.3389/fcvm.2022.880698

**Published:** 2022-06-28

**Authors:** Angelo Porfidia, Giulia Cammà, Nicola Coletta, Margherita Bigossi, Igor Giarretta, Andrea Lupascu, Giuseppe Scaletta, Enrica Porceddu, Paolo Tondi, Giovanni Scambia, Gabriella Ferrandina, Roberto Pola

**Affiliations:** ^1^Section of Internal Medicine and Thromboembolic Diseases, Fondazione Policlinico Universitario A. Gemelli Istituto di Ricerca e Cura a Carattere Scientifico (IRCCS), Rome, Italy; ^2^Università Cattolica del Sacro Cuore, Rome, Italy; ^3^Division of Population Health and Genomics, School of Medicine, Pat Macpherson Centre for Pharmacogenomics and Pharmacogenetics, University of Dundee, Dundee, United Kingdom; ^4^Department of Woman, Child, and Public Health, Fondazione Policlinico Universitario A. Gemelli IRCCS, Rome, Italy; ^5^Division of Angiology, Fondazione Policlinico Universitario A. Gemelli IRCCS, Rome, Italy

**Keywords:** central venous catheter (CVC), catheter-related thrombosis (CRT), gynecologic cancer, women, breast cancer, venous thromboembolism (VTE), anticoagulation, direct oral anticoagulant

## Abstract

**Background:**

Catheter-related thrombosis (CRT) of the upper extremities is a frequent complication among cancer patients that carry a central venous catheter (CVC) and may lead to pulmonary embolism (PE) and loss of CVC function. Despite its clinical impact, no anticoagulant treatment scheme has been rigorously evaluated in these patients. In addition, there is no proven evidence that direct oral anticoagulants (DOACs) are efficacious and safe in this setting because cancer patients with CRT of the upper extremities were not included in the clinical trials that led to the approval of DOACs for the treatment of cancer-associated venous thromboembolism (VTE).

**Methods:**

We performed a single center retrospective cohort study on women with gynecologic or breast cancer treated with either low-molecular-weight heparin, fondaparinux, or DOACs for CRT of the upper extremities. Only patients who received anticoagulation at the proper therapeutic dose and for at least 3 months were included in the analysis. Effectiveness was evaluated in terms of preservation of line function, residual thrombosis, and recurrence of VTE (including PE). Safety was evaluated in terms of death, major bleeding (MB), and clinically relevant non-major bleeding (CRNMB).

**Results:**

We identified 74 women who fulfilled the criteria to be included in the analysis. Of these, 31 (41.9%) had been treated with fondaparinux, 21 (28.4%) with enoxaparin, and 22 (29.7%) with the DOAC edoxaban. We found no differences between patients treated with the three different therapeutic approaches, in terms of preservation of line function, incidence of residual thrombosis, and VTE recurrence (including PE). Safety was similar as well, with no MBs recorded in any treatment group.

**Conclusion:**

These results, although retrospective and based on a relatively small sample size, indicate that, in women with gynecologic or breast cancer, CRT of the upper extremities may be treated with similar effectiveness and safety with fondaparinux, enoxaparin, and edoxaban. Further studies are needed to substantiate these findings.

## Introduction

Indwelling central venous catheters (CVC) are often used in cancer patients to provide a secure and reliable venous access and facilitate the administration of therapies. However, catheter-related thrombosis (CRT) is a common complication of indwelling CVC, especially in cancer patients, who are at high risk of venous thromboembolism (VTE) (1, 2). The reported incidence of CRT varies from around 5%, by considering only symptomatic events, up to an overall rate of 30%. CRT leads to pulmonary embolism (PE) in around 10–15% of cases and loss of line function in 10% of cases (3). Risk factors for CRT may be distinguished into patient-related, such as previous history of VTE, the presence of prothrombotic conditions, and the type of neoplasm, and catheter-related, such as the type of catheter and the insertion site. CRT may also be distinguished into early- and late-CRT, which occur within and beyond 30 days from CVC implantation, respectively. Early-CRT is usually considered a direct consequence of catheter placement, whereas late-CRT is generally independent from the insertion process (4). The gold standard for the diagnosis of CRT is venous Doppler ultrasound. Nonetheless, many cases are incidentally diagnosed on contrast-enhanced computed tomography (CT) scan. Indeed, in oncological patients, a number of asymptomatic CRT, that would otherwise remain undetected, is identified during CT scans performed for the follow-up of cancer (4).

Despite its epidemiological and clinical impact, no treatment scheme has been rigorously evaluated for CRT of the upper extremities in cancer patients. Indeed, evidence-based guidelines are lacking, and recommendations are mainly based upon indirect evidence from the experience with deep vein thrombosis (DVT) of the lower limbs. Thus, it is not surprising that treatment strategies in CRT are heterogeneous, and there are even patients who do not receive anticoagulation, but are instead treated by removal of the catheter alone (5). Consensus opinion however is for systemic anticoagulation, for a minimum of 3 months, and as long as the CVC remains *in situ* (6). Regarding the type of anticoagulation in patients with malignancy, the expert opinion is to use treatment doses of low-molecular weight heparin (LMWH), due to its superiority over vitamin K antagonists (VKAs) and the paucity of data on the use of DOACs in this setting (7). Indeed, cancer patients with CRT of the upper extremities were not included in the clinical trials that led to the approval of DOACs for the treatment of cancer-associated VTE. Nonetheless, DOACs are increasingly prescribed to these patients by many physicians in real-world clinical practice and there are sparse reports on their use (8–14).

We performed a single center retrospective cohort study to compare effectiveness and safety of different anticoagulants for the treatment of CRT of the upper extremities in women with either gynecologic or breast cancer.

## Materials and Methods

We carried out a systematic retrospective analysis of the electronic database of the Section of Internal Medicine and Thromboembolic Diseases (Thrombosis Outpatient Clinic) of the Fondazione Policlinico Universitario A. Gemelli IRCCS. The search was limited to the time period between 01 November 2019 and 31 October 2021.

First, we identified patients who had the following criteria: (i) female gender; (ii) diagnosis of active cancer, defined as cancer diagnosed or treated within the 2 years prior to VTE diagnosis, as previously established in the literature (15); and (iii) established diagnosis of CRT, personally confirmed by one of the study investigators by venous Doppler ultrasound of the upper limbs. Next, we selected patients who had been treated with either a LMWH, fondaparinux, or a DOAC, at the proper therapeutic dose, and at least for 3 months. To be included in the analysis, it was also necessary that the same medication had been administered to an individual patient throughout the 3 months of therapy. In the case of patients treated with DOACs, it was allowed that they had initially received parenteral anticoagulation. Nonetheless, we established that, in order to be included in the analysis, it was necessary that lead-in with heparin or fondaparinux prior to DOAC initiation had lasted no longer than 10 days. Finally, we included in the analysis only those patients for whom there was full availability of the follow-up data regarding the endpoints of interest of this study. Duration of follow-up was established as the time (in days) during which patients received the same anticoagulant therapy at the proper therapeutic dose. Exclusion criteria were: age <18 years and treatment with anticoagulants for reasons different from CRT (i.e., atrial fibrillation, or concomitant VTE in other sites).

Effectiveness endpoints were: preservation of line function, defined as the uninterrupted possibility of efficiently utilizing the CVC throughout follow-up, with no need of CVC removal and/or substitution; residual thrombosis after completion of 3 months of anticoagulant therapy, assessed by venous Doppler ultrasound; recurrence of symptomatic VTE (including PE), assessed by either venous Doppler ultrasound or CT pulmonary angiography, throughout follow-up. Safety endpoints were: death, major bleeding (MB), and clinically relevant non-major bleeding (CRNMB) throughout follow-up. MB and CRNMB were defined in accordance with the criteria established by the International Society on Thrombosis and Haemostasis (ISTH) (16). Briefly, MB was defined as overt bleeding resulting in a decrease of hemoglobin by at least 2 g/dL, requiring transfusion of at least two units of packed red blood cells, bleeding occurring in a critical site (intracranial, intraspinal, intraocular, retroperitoneal, etc.), or contributing to death. CRNMB was defined as overt bleeding not meeting MB criteria, which led to medical or surgical intervention, a hospital visit, or prompting a face to face evaluation. Effectiveness and safety events were inferred and adjudicated by the analysis of clinical charts by three independent investigators. Discrepancies were resolved by discussion between investigators.

Continuous variables are presented as mean ± standard deviation (SD), while categorical data are presented as absolute number (percentage). Days of follow-up are presented as median and interquartile range (IQR). Differences between groups were tested using one-way ANOVA, Chi-square, or Fisher's exact test, as appropriate. Statistical significance was reached when p-value was < 0.05. The analyses were performed by using StatCalc, version 1500.1.12 (64-Bit), Microsoft Excel ver. 14.6.3 (160329).

The study was approved by the Ethics Committee of the Fondazione Policlinico Universitario A. Gemelli IRCCS (Protocol number 49904/18).

## Results

We found 74 women who fulfilled the criteria to be included in the study. Among these patients, 21 (28.4%) had received parenteral anticoagulation with enoxaparin, 31 (41.9%) parenteral anticoagulation with fondaparinux, and 22 (29.7%) oral anticoagulation with edoxaban (preceded by a mean lead-in with heparin or fondaparinux of 6.1 ± 0.4 days).

Table 1 presents a comparison of the demographic and clinical characteristics of the patients, according to the treatment group. There were no statistically significant differences between the three groups in terms of age, type of neoplasm, site of CRT, and type of CVC. Median follow-up time was 162 days [IQR 102—256] in the edoxaban group, 180 days [IQR 125—271] in the enoxaparin group, and 164 days [IQR 118—192] in the fondaparinux group. Also this difference was not statistically significant.

**Table 1:** Comparison between patients treated with edoxaban, enoxaparin, and fondaparinux.

**Characteristics of patients**	**Edoxaban** **(*n* = 22)**	**Enoxaparin** **(*n* = 21)**	**Fondaparinux** **(*n* = 31)**	***P***
Age (years ± SD)	57.6 ± 12.7	55.0 ± 13.1	54.7 ± 12.6	0.69
Type of cancer	
Ovarian cancer, *n* (%)	9 (40.9)	11 (52.4)	9 (29.0)	0.23
Endometrial cancer, *n* (%)	4 (18.2)	2 (9.5)	8 (25.8)	0.33
Cervical cancer, *n* (%)	3 (13.6)	3 (14.3)	2 (6.4)	0.59
Breast cancer, *n* (%)	6 (27.2)	5 (23.8)	12 (38.7)	0.47
Stage of cancer				
Localized, *n* (%)	7 (31.8)	6 (28.6)	7 (22.6)	0.96
Metastatic, *n* (%)	15 (68.2)	15 (71.4)	24 (77.4)	0.96
Active chemotherapy, *n* (%)	20 (90.9)	21 (100.0)	29 (93.5)	0.76
Site of CRT	
Jugular vein, *n* (%)	0 (0.0)	4 (19.0)	3 (9.7)	0.10
Subclavian vein, *n* (%)	4 (18.2)	6 (28.6)	6 (19.3)	0.63
Brachiocephalic vein, *n* (%)	0 (0.0)	1 (4.8)	1 (3.2)	0.61
Axillary vein, *n* (%)	6 (27.3)	6 (28.6)	13 (41.9)	0.45
Basilic vein, *n* (%)	10 (45.4)	3 (14.2)	7 (22.6)	0.06
Brachial vein, *n* (%)	2 (9.1)	1 (4.8)	1 (3.2)	0.64
Early thrombosis, *n* (%)	13 (59.1)	10 (47.6)	15 (48.4)	0.68
Late thrombosis, *n* (%)	9 (40.9)	11 (52.4)	16 (51.6)	0.68
Type of CVC	
PICC, *n* (%)	7 (31.8)	3 (14.3)	9 (29.0)	0.36
Port-a-cath, *n* (%)	15 (68.2)	18 (85.7)	22 (71.0)	0.36
Laboratory data before initiating anticoagulation	
Hemoglobin, (g/dL ± SD)	11.8 ± 1.5	10.9 ± 1.7	12.2 ± 1.4	0.01
Creatinine (mg/dL ± SD)	0.8 ± 0.2	0.7 ± 0.2	0.7 ± 0.2	0.15
Platelets (cells ×10^9^/L ± SD)	283.9 ± 124.9	329.8 ± 137.2	289.1 ± 126.8	0.43
Days of follow-up (median [IQR])	162 [102–256]	180 [125–271]	164 [118–192]	0.48

*CRT, catheter-related thrombosis; CT, computed tomography; CVC, central venous catheter; IQR, interquartile range; PICC, peripherally inserted central catheter; SD, standard deviation*.

Effectiveness and safety outcomes are presented in Table 2. Residual thrombosis at 3 months was found in 1 of the patients treated with edoxaban (4.5%), 1 of the patients treated with enoxaparin (4.8%), and 5 of the patients treated with fondaparinux (16.0%). These differences were not statistically significant. Likewise, there was no statistically significant difference between groups in terms of preservation of line function (which was achieved in 100.0% of patients) and recurrence of VTE (which was 0.0% in all groups). Regarding safety endpoints, no deaths (0.0%) were recorded in any of the groups during follow-up. There were no MBs. There were 2 CRNMBs in the group of patients treated with enoxaparin (9.5%). There was 1 CRNMB in the group of patients treated with fondaparinux (3.2%). No CRNMBs were found in the group of patients treated with edoxaban (0.0%).

**Table 2:** Comparison of effectiveness and safety between patients treated with edoxaban, enoxaparin, and fondaparinux.

	**Edoxaban** **(*n* = 22)**	**Enoxaparin** **(*n* = 21)**	**Fondaparinux** **(*n* = 31)**	***P***
Residual thrombosis, *n* (%)	1 (4.5)	1 (4.8)	5 (16.0)	0.25
Preservation of line function, *n* (%)	22 (100.0)	21 (100.0)	31 (100.0)	na
Recurrent VTE, *n* (%)	0 (0.0)	0 (0.0)	0 (0.0)	na
MB, *n* (%)	0 (0.0)	0 (0.0)	0 (0.0)	na
CRNMB, *n* (%)	0 (0.0)	2 (9.5)	1 (3.2)	0.27
Death, *n* (%)	0 (0.0)	0 (0.0)	0 (0.0)	na

*CRNMB, clinically relevant non-major bleeding; MB, major bleeding; na, not available; VTE, venous thromboembolism*.

## Discussion

The best therapeutic strategy for thrombosis associated with cancer is object of intense debate. Recent randomized clinical trials have demonstrated that DOACs are an effective and safe therapeutic alternative to LMWH in cancer patients with VTE (15, 17, 18). However, this is true and well established only for patients with DVT of the proximal veins of the lower limbs and/or PE, while level of evidence is very low in the case of thrombosis involving other venous sites, such as the upper extremities (19). Things are even more confused when the thrombosis of the upper extremities is related to the presence of a CVC. Indeed, cancer patients with DVT of the upper extremities were not included in the DOAC trials mentioned above (15, 17, 18). There was a small number of patients with upper-extremity DVT (46/300) in the ADAM VTE trial – a study that was published before the Caravaggio trial and had a similar design (apixaban vs. LMWH) but smaller patient sample – but it is unknown how many of these thromboses were catheter-related (20). To date, the only information available on DOACs in cancer patients with CRT derive from small cohort studies that mainly used rivaroxaban (8–14). However, only two of these studies were specifically focused on cancer patient. In particular, one was a retrospective analysis on 83 cancer patients with catheter-related VTE treated with rivaroxaban (10). In 3.6% of patients the catheter was removed due to the line dysfunction and the major bleeding risk was 2.4% (10). The other study—named CATHETER 2—included 70 patients with active malignancy and symptomatic catheter-related VTE who were treated with rivaroxaban for 12 weeks (9). The preservation of line function was achieved in 100% of patients at 12 weeks, while the rate of recurrent VTE was 1.4% with a risk of major bleeding of about 10% (9).

In this scenario, our study is novel for several reasons. First, it only includes cancer patients. Second, it is specifically focused on women with gynecologic or breast cancer. Third, it provides a comparison between a DOAC (edoxaban) and parenteral anticoagulation in patients with upper extremity-CRT. Our results suggest that edoxaban—which is already approved for the treatment of proximal lower-limb DVT and EP in cancer patients—is effective and safe also for CRT of the upper extremities, with preservation of line function and resolution of thrombosis in almost the totality of patients and no evidence of MB and CRNMB. These findings are consistent with the results of a recent meta-analysis published by our group, which has shown that recurrent VTE, MB, and CRNMB are not significantly different in patients with upper extremity DVT who received different types of anticoagulant treatments, even if they are affected by cancer (21). The fact that our data have been obtained in a specific population of women with gynecologic cancer is important, because it provides novel information on a category of patients affected by cancers that are frequently associated with VTE and because CRT often occurs in women receiving chemotherapy for cancer (22).

This study has limitations. It is a retrospective analysis, the patient sample is small, and prescription bias might exist between patients treated with edoxaban vs. those treated with parenteral anticoagulation, although they did not differ in terms of age, type of cancer, type of CVC, site of CRT, and duration of treatment and follow-up.

In conclusion, our data indicate that, in women with gynecologic or breast cancer, CRT of the upper extremities may be treated with similar effectiveness and safety with fondaparinux, enoxaparin, and edoxaban, supporting the hypothesis that DOACs might be an alternative to parenteral anticoagulation in this set of patients. Prospective studies and a larger sample size are needed to confirm these findings.

## Data Availability Statement

The raw data supporting the conclusions of this article will be made available by the authors, without undue reservation.

## Ethics Statement

The studies involving human participants were reviewed and approved by Ethics Committee of the Fondazione Policlinico Universitario A. Gemelli IRCCS. Written informed consent for participation was not required for this study in accordance with the national legislation and the institutional requirements.

## Author Contributions

AP, GC, NC, IG, GF, and RP contributed to conception and design of the study. AP, GC, NC, IG, MB, AL, GiuS, and EP were in charge of patients' care. GC, NC, MB, and IG organized the database. GC and NC performed statistical analyses. AP, GC, and NC wrote the first draft of the manuscript. PT, GioS, GF, and RP supervised the work and edited the manuscript. All authors contributed to manuscript revision, read, and approved the submitted version.

## Conflict of Interest

The authors declare that the research was conducted in the absence of any commercial or financial relationships that could be construed as a potential conflict of interest.

## Publisher's Note

All claims expressed in this article are solely those of the authors and do not necessarily represent those of their affiliated organizations, or those of the publisher, the editors and the reviewers. Any product that may be evaluated in this article, or claim that may be made by its manufacturer, is not guaranteed or endorsed by the publisher.
